# CB1 Cannabinoid Receptor Signaling and Biased Signaling

**DOI:** 10.3390/molecules26175413

**Published:** 2021-09-06

**Authors:** Luciana M. Leo, Mary E. Abood

**Affiliations:** Center for Substance Abuse Research, Lewis Katz School of Medicine, Temple University, Philadelphia, PA 19140, USA; luciana.leo@temple.edu

**Keywords:** cannabinoid, CB1, biased signaling, functional selectivity, G-protein, β-arrestin

## Abstract

The CB1 cannabinoid receptor is a G-protein coupled receptor highly expressed throughout the central nervous system that is a promising target for the treatment of various disorders, including anxiety, pain, and neurodegeneration. Despite the wide therapeutic potential of CB1, the development of drug candidates is hindered by adverse effects, rapid tolerance development, and abuse potential. Ligands that produce biased signaling—the preferential activation of a signaling transducer in detriment of another—have been proposed as a strategy to dissociate therapeutic and adverse effects for a variety of G-protein coupled receptors. However, biased signaling at the CB1 receptor is poorly understood due to a lack of strongly biased agonists. Here, we review studies that have investigated the biased signaling profile of classical cannabinoid agonists and allosteric ligands, searching for a potential therapeutic advantage of CB1 biased signaling in different pathological states. Agonist and antagonist bound structures of CB1 and proposed mechanisms of action of biased allosteric modulators are used to discuss a putative molecular mechanism for CB1 receptor activation and biased signaling. Current studies suggest that allosteric binding sites on CB1 can be explored to yield biased ligands that favor or hinder conformational changes important for biased signaling.

## 1. Introduction

The cannabinoid receptor type 1 (CB1) is a class A G-protein coupled receptor (GPCR) that was first discovered as the main target for Δ^9^-tetrahydrocannabinol (THC), the psychoactive compound in *Cannabis*. CB1 was first described in rat [1,2] and later cloned from a human brain cDNA library [3]. At the protein level, rat and human CB1 share 97% sequence identity, with only two amino acid substitutions within the transmembrane domains. Of these, one is found on the extracellular (EC) end of transmembrane helix 2 (TMH2)—position 2.62 in Ballesteros–Weinstein nomenclature [4], Ile175 in human and Val176 in rat—and one on the EC end of TMH3—position 3.22, Arg186 in human and Pro187 in rat. Interestingly, the C-terminal tail of CB1 forms an extra α-helix between residues Ala440 and Met461 (amino acid numbers for human CB1), termed Helix 9 (Hx9), that associates with the plasma membrane.

In addition to THC, other exogenous ligands for CB1 have been described. Notably, THC analogs and other synthetic cannabinoids are widely used as CB1 agonists, such as HU-210, CP55940, and WIN55212 [5,6,7]. Endogenous ligands for CB1 are derived from arachidonic acid, which is metabolized by diacylglycerol lipase into 2-arachidonoylacylglycerol (2-AG) and by N-acyl-phosphatidylethanolamine-hydrolyzing phospholipase D into anandamide (AEA) [8,9,10]. AEA and 2-AG are primarily degraded by fatty acid amide hydrolase and monoacylglycerol lipase, respectively [11]. However, 2-AG and AEA also bind to other targets, such as the CB2 receptor and transient receptor potential vanilloid 1 (TRPV1) [11]. These endogenous ligands, their receptors, and their synthesis and degradation enzymes form the endocannabinoid system [11].

Here, we will review the role of CB1 in physiological and pathological conditions and explore its various signaling mechanisms. CB1 has been investigated as a source of biased signaling, a process by which a given ligand can preferentially elicit signaling via one signal transducer to the detriment of another [12]. However, the physiological role of CB1-biased signaling is poorly understood. Therefore, studies that suggest a therapeutic advantage for CB1 biased ligands will be discussed. Finally, considering the solved molecular structures of agonist bound CB1, along with the proposed mechanisms of action of certain biased allosteric modulators, we will analyze the potential molecular mechanism of CB1 biased signaling.

## 2. The CB1 Receptor

### 2.1. Therapeutic Potential

Dysregulation of the endocannabinoid system in physiological aging and in brain pathologies along with the prevalence of CB1 in a variety of CNS circuits make it an attractive target for the treatment of multiple neurological conditions. In fact, *Cannabis* and cannabinoid formulations are already approved for certain medicinal uses in several countries and in most US states. Dronabinol and nabilone are synthetic THC analogs approved by the US Food and Drug Administration as antiemetics and orexigenics for patients undergoing chemotherapy and patients with acquired immunodeficiency syndrome. Nabiximols (Sativex^®^) are *Cannabis* extracts containing THC and cannabidiol at a near 1:1 ratio approved in the United Kingdom, Spain, Brazil, Colombia, Chile, Australia, among several countries, for mitigation of symptoms, including spasticity, of treatment-resistant multiple sclerosis. In the United States, Sativex^®^ is currently under investigation in a phase 3 clinical trial for the treatment of neuropathic pain (NCT00711880) with promising preliminary results [13]. There are also several currently active clinical trials investigating the efficacy of medicinal *Cannabis* use in the treatment of acute and chronic pain. The analgesic properties of cannabinoids are well known, and enhancing CB1 activity has been proposed as a treatment for various forms of pain [14] due to its ability to suppress nociception at dorsal root ganglia [15,16], spinal cord [17,18,19], and the descending pain modulatory system, such as in the periaqueductal gray (PAG) [20,21,22].

Although cannabinoid use is generally associated with cognitive impairment [23], a recent study showed that, while in young mice a chronic low dose THC treatment acts through CB1 to impair memory, it has the opposite effect in aged mice [24]. This result, along with findings of reduced CB1 expression and function in aged mice [25] and of early onset cognitive dysfunction in mice with CB1 deletion [26], suggests that CB1 agonists may have a beneficial effect in the treatment of age-related cognitive impairment.

CB1 agonists have also been shown to reduce anxiety-like behavior [27,28] and depressive-like behavior [29,30] in preclinical models, showing promise for the treatment of generalized anxiety and major depression disorders. The anxiolytic effect of cannabinoids, along with their negative modulation of hypothalamus–pituitary–adrenal axis activity mediated stress responses and facilitation of extinction learning in fear memory, led cannabinoid agonists to be investigated in the treatment of posttraumatic stress disorder (PTSD). In this context, positive results have been reported from CB1 and CB2 agonists in preclinical models [31], and a current phase 2 clinical study is underway to investigate the effect of *Cannabis* on symptoms of PTSD in war veterans (NCT02759185). Further, the anticonvulsant action of cannabinoids in preclinical models makes CB1 a possible target for the treatment of epilepsy [32,33].

Neuroprotection has been suggested as a function of the endocannabinoid system, and findings that CB1 agonists protect against cerebral ischemia and that CB1 deletion enhances the severity of ischemia–reperfusion injury in mice [34,35,36] suggest that it could also be targeted for the treatment of stroke. Finally, there is evidence that CB1 activity is beneficial for the treatment of Huntington’s disease (HD), a genetic neurodegenerative disorder marked by expression of mutant Huntingtin (mHTT) protein with polyglutamine repeats, which forms aggregates that lead to striatal neurodegeneration and progressive motor dysfunction [37]. Loss of CB1 receptors in basal nuclei was reported in HD mouse models [38,39] and in the brains of HD patients [40]. These findings suggest that CB1 function is impaired in HD, and therefore, restoring CB1 signaling could have a beneficial effect in the treatment of HD. Indeed, Chairlone et al. found that deletion of CB1 receptors from glutamatergic corticostriatal neurons exacerbates striatal neuron cell death and motor dysfunction in a mouse model of HD [41]. Therefore, CB1 agonists may mitigate HD progression and motor symptoms.

### 2.2. CB1 Physiology

CB1 is the main endocannabinoid system GPCR in the nervous system and is one of the most highly expressed GPCRs in the central nervous system (CNS). Neurons are the primary source of CB1 expression in the CNS, where a high density of CB1 is found in axons, especially at presynaptic terminals [42]. In presynaptic terminals, endocannabinoids act as retrograde neuromodulators, that is, synaptic transmission triggers endocannabinoid synthesis at the postsynaptic terminal, which activate presynaptic CB1 receptors that, in turn, inhibit neurotransmitter release [43]. Since CB1 is found in both GABAergic and glutamatergic synapses, endocannabinoids induce short-term synaptic plasticity via depolarization-induced suppression of inhibition (DSI in GABAergic terminals) or depolarization-induced suppression of excitation (DSE in glutamatergic terminals) [44]. However, CB1 does not act only in presynaptic terminals but also regulates somatodendritic excitability, such as in low-threshold spiking cortical interneurons, where 2-AG promotes slow self-inhibition [45]. A putative role for CB1 in neuronal mitochondria has been proposed, where it could contribute to suppression of neurotransmitter release by negatively regulating mitochondrial respiration and adenosine triphosphate (ATP) generation [46]. To a lower extent, CB1 is expressed in astrocytes, where it regulates gliotransmitter release, glucose metabolism, and the release of inflammatory mediators [47,48,49,50]. CB1 is not found at the protein level in resting microglia but has been detected in activated microglial cells in primary cultures from mollusk, mouse, and rat but not human tissue [51]. Additionally, CB1 is found in neurons of the dorsal root ganglia (DRG), in peripheral nerve terminals, and in neurons of the enteric nervous system [52]. At low levels, CB1 is also expressed in some peripheral tissues, such as adipose tissue, testis, prostate, adrenal glands, thymus, bone marrow, and heart [42].

### 2.3. Toxicity and Adverse Effects

Cannabinoids are generally well tolerated; however, acute and chronic toxicity is known to occur after consumption of *Cannabis* or, more frequently, synthetic cannabinoids. In the CNS, cannabinoids can induce cognitive and psychomotor impairment. In more severe cases, and especially with synthetic cannabinoids, agitation and acute psychosis may occur [53]. Regulation of neurotransmitter release by CB1 receptors is likely responsible for these effects. Overactivation of peripheral CB1 can also contribute to the development and progression of cardiovascular and metabolic diseases. Notably, the endocannabinoid system can affect cardiovascular function in a complex manner. CB1 activation reduces cardiac contractility likely via sympathetic inhibition and reduced Na^+^ and Ca^2+^ influx in myocytes [54]. Further, CB1 activation causes hypotension in healthy individuals, but CB1 antagonism reduced blood pressure in obese and diabetic patients with hypertension [54]. Similarly, CB1 activation may exacerbate myocardial injury in the context of cardiac pathology [55]. CB1 can also contribute to diet-induced obesity. In addition to regulating feeding behavior in the CNS [56], peripheral CB1 enhances lipogenesis [57,58,59], inhibits lipolysis [59,60,61], and promotes leptin resistance [62,63,64]. Peripheral CB1 also promotes the development of nonalcoholic fatty liver disease [65,66,67], pancreatic β-cell death, and peripheral insulin resistance [68,69,70]. Therefore, therapies aiming at CB1 agonism may not be suitable for patients that already suffer from cardiovascular and metabolic diseases. In these contexts, CB1 antagonism and inverse agonism could be viable therapeutic strategies. However, the CNS effects of CB1 blockade have proven to be hazardous, as exemplified by the Rimonabant clinical trials, a CB1 inverse agonist that promoted depression and suicide [71,72]. Peripherally restricted CB1 antagonists and inverse agonists are currently being pursued to avoid CNS effects [59,60,63,67,70].

## 3. CB1 Mechanism of Activation

X-ray crystal structures of inactive CB1 bound to an antagonist/inverse agonist [73,74], canonical active CB1 bound to a potent agonist [75], and cryoelectron microscopy (cryo-EM) structures of CB1 in complex with heterotrimeric G_i_ protein [76,77] have been determined. In the inactive structure studies, the ionic lock between Arg214^3.50^ and Asp338^6.30^ (Ballesteros–Weinstein nomenclature [4] in superscript) was present in CB1, and the antagonist/inverse agonist compounds were deduced to enter the binding pocket via a gap between TMH1 and TMH7 [73]. In the active state structure study, novel washout resistant agonists were generated to enable crystallography. These were of similar potency and efficacy to CP55940 in a cAMP inhibition assay [75], but non-Gα_i/o_ signaling, receptor internalization, or β-arrestin recruitment were not evaluated. The ligand-binding pocket was formed mainly by hydrophobic interactions with residues on extracellular loop 2 (ECL2), TMH3, TMH5, TMH6, and TMH7 [74,75], apart from a hydrogen bond formed between the phenolic hydroxyl of the agonist AM11542 and Ser383^7.39^. Importantly, a previous study showed that mutating Ser383^7.39^ to Ala resulted in severely reduced binding of several CB1 ligands [78], further supporting the role of Ser383^7.39^ in ligand-binding interactions. Comparing the structures of antagonist-bound and agonist-bound CB1 revealed important features that likely participate in the molecular mechanism of receptor activation. The most noticeable conformational change in the transmembrane helices is the outward movement of the intracellular (IC) domain of TMH6. In the CWXP motif, a “twin toggle switch” mechanism is formed between Trp356^6.48^ and Phe200^3.36^. In the inactive state, the side chains of Phe200^3.36^ and Trp356^6.48^ point away and toward the ligand-binding pocket, respectively, forming an aromatic stacking interaction that maintains the inactive state. Upon agonist binding, the rotation of TMH3 causes the Phe200^3.36^ side chain to flip, facing the binding pocket and disrupting the interaction with Trp356^6.48^. Now released, Trp356^6.48^ rotates inward, which results in the relaxation of the kink at Pro358^6.50^, causing TMH6 to straighten, moving its IC end away from the receptor core [75,76]. This “twin toggle switch” mechanism was previously demonstrated using mutagenesis and molecular dynamics (MD) simulations [79] and was confirmed by the crystal structure. Conformational changes important to receptor activation also occur in the DRY motif, where Arg214^3.50^ adopts an extended conformation, leading to disruption of the hydrogen bonding network with Asp213^3.49^ and Asp338^6.30^ (ionic lock). With the ionic lock broken, TMH6 moves outward, exposing sites for interaction with the G-protein. In the NPXXY motif, TMH7 unwinds around Tyr397^7.53^. Further interactions formed by amino acids in this motif are not shown. Although the crystal structure elucidated many important features of agonist binding and molecular mechanisms of activation, there is an issue with the receptor used in the study. Four amino acid mutations (T210A, E273K, T283V, R340E) were introduced to improve expression and thermostability, thus allowing crystallography to be performed. This could have an impact on the overall structure of the activated receptor. In fact, the T210A mutation reduced cAMP inhibition in response to three different agonists, and Hua et al. state that the modified receptor construct cannot induce signaling [75,80].

Nonetheless, a cryo-EM structure of human CB1 bound to the highly potent agonist MDMB-FUBINACA and in complex with G_i_ showed that the two structures highly match, with a broad overlap in the ligand-binding site and “twin toggle switch” mechanism [76]. Differences were found in a more extended outward movement of the IC end of TMH6 and rotation of Arg214^3.50^ toward the α5-helix of the Gα_i_ protein. Further, they found a weaker interaction between the intracellular loop 2 (ICL2) of CB1 and the Ras domain of the Gα_i_, which could explain the G-protein coupling promiscuity observed with CB1 [76]. These studies provided much valuable information on the mechanism behind G-protein signaling at CB1. However, in the crystal structures, the agonist’s ability to promote β-arrestin recruitment is unknown. Further, the receptor was truncated at the C-terminus for crystallization, which precludes β-arrestin binding. In the cryo-EM structure, the receptor is stabilized by forming a complex with G_i_, so this conformation is most likely to resemble the one responsible for G-protein-biased signaling. Therefore, the active CB1 structures available to date do not provide clues to a molecular mechanism behind β-arrestin-biased signaling.

Arrestins bind to GPCRs in two locations, the phosphorylated C-terminus and the cytoplasmic end of the activated GPCR transmembrane core [81]. One of these β-arrestin-GPCR core interactions occurs with Arg214^3.50^ in the highly conserved DRY motif [82]. A mutational study focused on the role of the DRY motif in CB1 G-protein signaling and β-arrestin recruitment [83]. They found that mutating both Arg214^3.50^ and Tyr215^3.51^ to Ala (DAA) yielded a CB1 receptor with a G-protein-biased signaling profile. Although G-protein signaling was partially reduced, β-arrestin recruitment was eliminated. In contrast, mutating Asp213^3.49^ and Arg214^3.50^ to Ala (AAY) yielded a CB1 receptor with a β-arrestin-biased signaling profile. While G-protein signaling was reduced, β-arrestin recruitment was enhanced. Both mutated receptors also have increased constitutive activity. These mutations impacted both G-protein and β-arrestin signaling, consistent with the roles of the DRY motif in the interaction between the α5-helix of the G-protein [76] and the finger loop of β-arrestin [82]. However, it is possible that increases in β-arrestin recruitment are due to impaired G-protein coupling and reduced competition for GPCR binding. Nonetheless, the intramolecular interactions that promote β-arrestin-biased signaling at the CB1 receptor remain elusive.

Biophysical studies of other GPCRs show that while G-protein-biased ligands induce movement of TMH6, β-arrestin-biased ligands favor movement of TMH7 [84,85,86]. Unfortunately, these studies cannot identify precise structural modifications or intramolecular interactions. Crystal structures of the 5-HT2B receptor bound to the β-arrestin-biased ligand ergotamine [87] and visual arrestin bound Rhodopsin [88] showed a reduced movement of TMH6, compared to the canonical G-protein active state and structural modifications on TMH7 and Hx8. Although the structure of β-arrestin bound CB1 has not been solved yet, the studies from other class A GPCRs indicate that a similar molecular mechanism involving TMH7/Hx8 regulates β-arrestin-biased signaling in CB1.

## 4. CB1 Signaling

### 4.1. G-Proteins

**Figure 1:**
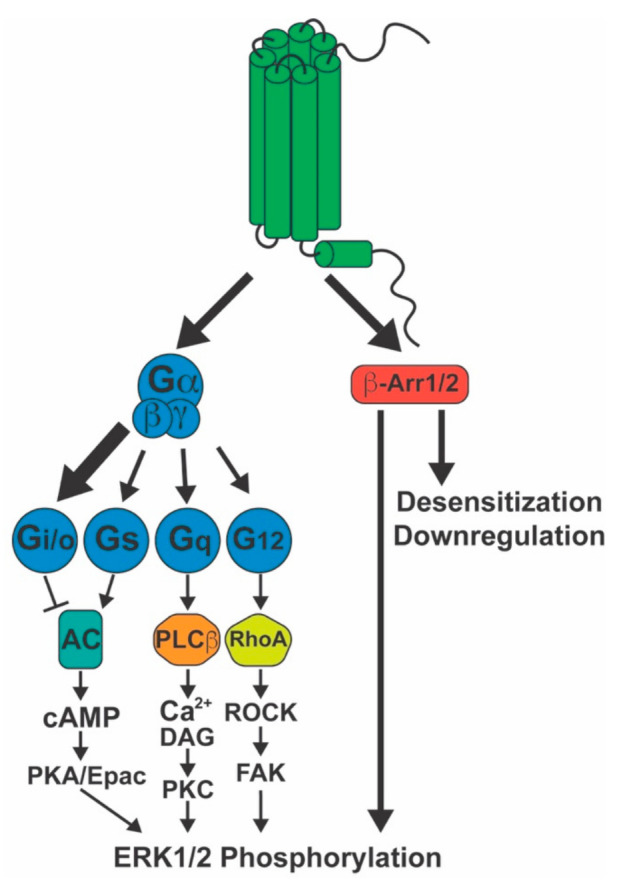
Signaling activated by the CB1 receptor. The CB1 receptor primarily couples to G_i/o_ proteins (large arrow), but also to G_s_, G_q/11,_ and G_12/13_ (smaller arrows) to a lower extent. CB1 also recruits both β-arrestin1 and β-arrestin2. These proteins mediate receptor desensitization, endocytosis, and pERK1/2 signaling. The latter can also be induced by downstream signaling events stemming from the G-protein pathways. AC: adenylyl cyclase; β-Arr1/2: β-arrestin1/2; cAMP: cyclic adenosine monophosphate; DAG: diacylglycerol; Epac: exchange protein directly activated by cAMP; ERK1/2: extracellular-signal-regulated kinase ½; FAK: focal adhesion kinase; PKA: protein kinase A; PKC: protein kinase C; ROCK: Rho-associated protein kinase.

Canonical GPCR signaling depends on the coupling to heterotrimeric G-proteins, composed of α, β, and γ subunits. These are classified according to the type of Gα subunit, which will activate or inhibit specific second messengers, leading to different downstream signaling events. Gα_s_ proteins stimulate the activity of adenylyl cyclase (AC), enhancing cAMP levels, while Gα_i/o_ proteins inhibit AC, suppressing cAMP production (Figure 1). This second messenger binds to and activates protein kinase A (PKA), the exchange protein directly activated by cAMP (Epac) and cyclic nucleotide-gated ion channels, stimulating intracellular signaling cascades that regulate a variety of essential cellular functions, such as metabolism, gene expression, cell growth and differentiation, apoptosis and neurotransmission [89]. On the other hand, Gα_q/11_ proteins stimulate the activity of phospholipase C (PLC) β, an enzyme that catalyzes the hydrolysis of phosphatidylinositol 4,5-biphosphate (PIP_2_), releasing diacylglycerol (DAG) and inositol 1,4,5-triphosphate (IP_3_). The latter binds to IP_3_ receptors in the endoplasmic reticulum (ER), releasing Ca^2+^ from the ER to the cytoplasm. The increase in cytosolic calcium levels leads to activation of various signaling cascades, including activation of protein kinase C (PKC) [90]. Gα_12/13_ proteins recruit RhoGEFs to the membrane, leading to the activation of RhoA, which, in turn, activates Rho-associated protein kinase (ROCK). ROCK catalyzes the phosphorylation of focal adhesion kinase (FAK), stimulating the formation of actin stress fibers. ROCK also inhibits myosin light chain phosphatase, promoting cell contractility, and activates serum response factors [91]. These signaling pathways are also known to modulate the activation of extracellular signal-regulated kinase homologs 1 and 2 (ERK1/2).

Presynaptic membrane CB1 receptors induce DSI and DSE through suppression of neurotransmitter release via G-protein activity. CB1 mainly couples to G_i/o_ proteins (Figure 1) [5], leading to reduced cAMP levels via Gα_i/o_ and inhibition of voltage-gated Ca^2+^ channels via Gβγ, both of which suppress neurotransmitter release [44]. Although G_i/o_ proteins account for most of CB1-stimulated G-protein activity, low efficacy coupling to Gα_s_ (Figure 1), about 10% of total Gα_i/o_ coupling, has been described in N18TG2 neuroblastoma cells in response to CP55,940 [92]. As a result, in conditions where Gα_i/o_ proteins are suppressed, such as under Pertussis toxin (PTx) treatment, CB1 agonists stimulate cAMP accumulation [93,94,95]. Under physiological conditions, however, since Gα_i/o_ coupling is much more significant compared to Gα_s_, the net effect of CB1 agonists is to suppress AC activity and cAMP production. Coupling to Gα_q/11_ has been reported in human embryonic kidney (HEK293) cells transfected with CB1 receptor (Figure 1), but WIN55212-2 was the only agonist capable of eliciting Gα_q/11_ mediated Ca^2+^ signaling, suggesting ligand specificity for this response [96]. CB1 Gα_12/13_ coupling has been suggested (Figure 1) due to AEA-induced B103 neuroblastoma cell rounding, which was found to be dependent on ROCK and independent of Gα_i/o_ [97]. An evaluation of [^35^S]GTPγS binding in N18TG2 cells demonstrated that Gα_12/13_ activity accounts for about 7 to 10% of G-protein activity in unstimulated and CP55940 stimulated cells, respectively [98]. Further, WIN55212-2 was found to induce growth cone retraction in primary hippocampal neurons, and this effect was disrupted by suppression of Gα_12_ and Gα_13_ expression using small interfering ribonucleic acid (siRNA), which suggests that cannabinoid receptors induce Gα_12/13_ to regulate neurite growth [99]. Studies supporting Gα_12/13_ signaling by CB1 remain limited, and this pathway, therefore, still requires further characterization.

### 4.2. β-Arrestins

Not unlike other class A GPCRs, CB1 is capable of recruiting β-arrestins. Ligand-induced interaction with both β-arrestin1 and β-arrestin2 has been demonstrated [100,101]. These are known to induce receptor desensitization and internalization. Therefore, chronic exposure to cannabinoids leads to tolerance and downregulation of CB1 receptor activity in the brain [102,103,104,105], which could underlie *Cannabis* dependence. In addition, β-arrestin recruitment can promote ERK1/2 phosphorylation (pERK1/2) via the scaffolding of mitogen-activated protein kinases [106]. Although pERK1/2 is induced by either heterotrimeric G-proteins or β-arrestins (Figure 1), these responses differ in magnitude, kinetics, and likely physiological function. The G-protein-mediated pERK response was found to be strong, fast, and transient, while the β-arrestin-mediated pERK response is of lower magnitude, slow, and longer lasting [107]. Further, the subcellular location of pERK differs depending on the originating signal. G-protein mediated pERK1/2 is largely translocated to the nucleus, where it promotes gene transcription and cell proliferation. Conversely, β-arrestin-induced pERK1/2 concentrates on endosomes, inhibiting gene transcription and phosphorylating cytoplasmic substrates that regulate protein translation, cytoskeleton dynamics, apoptosis, cell migration, and cross talk with other signaling cascades [107,108,109,110].

Interestingly, β-arrestin deletion studies have shown that β-arrestin recruitment and signaling can have different effects on cannabinoid-induced behaviors. When administered systemically, CB1 receptor agonists produce four typical behaviors that are used in a battery of tests for preclinical models to assess cannabinoid response, known as the cannabinoid tetrad—analgesia, hypothermia, catalepsy, and hypolocomotion [111]. Cannabinoid tetrad tests were used to investigate cannabinoid responsiveness in mice lacking either β-arrestin1 or β-arrestin2. Mice with deletion of β-arrestin1 showed reduced analgesia and hypothermia in response to CP55940 under acute treatment but not in response to THC. This occurred despite the fact that β-arrestin1 knockout (KO) enhanced [^35^S]GTPγS binding induced by CP55,940 in cortex membranes, indicating a loss of G-protein desensitization [105]. This finding suggests that receptor desensitization, pERK1/2 signaling, or both β-arrestin1 functions together contribute to antinociception in mice. In contrast, antinociception or hypothermia induced by acute CP55940 treatment was not influenced by deletion of β-arrestin2, while THC-mediated antinociception and hypothermia were increased in β-arrestin2 KO mice [112]. Interestingly, a follow-up study found that despite increasing cannabinoid radioligand binding and availability in whole-brain P2 subcellular fraction—crude synaptosomes—β-arrestin2 KO actually decreased basal and agonist-stimulated [^35^S]GTPγS binding in hippocampus and cortex, while [^35^S]GTPγS binding in the cerebellum was unchanged [104]. This finding suggests that the increased antinociception and hypothermic effects of THC in β-arrestin2 KO mice are not due to increased G-protein signaling but may reflect a role of β-arrestin1 in mediating these cannabinoid-induced behaviors and a negative regulatory role for β-arrestin2 on the effects of β-arrestin1 signaling by cannabinoid receptors. Unfortunately, the authors did not evaluate G-protein signaling in the hypothalamus, midbrain, and spinal cord, where CNS regions involved in these responses are found. Taken together, these studies show that under acute treatment, β-arrestin1, and β-arrestin2 can have diverging effects on cannabinoid-induced antinociception and hypothermia. Catalepsy and hypolocomotion were not investigated in these studies; therefore, the role of β-arrestin1 and β-arrestin2 in these behaviors remains unknown. In addition, cannabinoid ligands, under certain conditions, may preferentially recruit β-arrestin1 or β-arrestin2, with CP55940 favoring β-arrestin1 and THC favoring β-arrestin2 [105,112].

Another study investigated the role of β-arrestin2 deletion on chronic cannabinoid exposure and tolerance development and found that β-arrestin2 downregulates CB1 receptor activity in a brain region-specific manner [103]. In accordance with Breivogel et al. [112], this study found that β-arrestin2 KO increased antinociception and hypothermia in response to acute THC treatment; however, no difference was found in cannabinoid-induced G-protein activity in CNS regions associated with antinociception, i.e., PAG and spinal cord, or hypothermia—the preoptic area of the hypothalamus. In contrast, the catalepsy response to acute THC was not affected by the β-arrestin2 deletion. After repeated THC administration, on the other hand, wild-type (WT) and β-arrestin2 KO mice developed different degrees of tolerance to THC antinociception, hypothermia, and catalepsy [103]. Although both genotypes develop a similar level of tolerance to hypothermia, tolerance to antinociception was attenuated in β-arrestin2 KO mice. Correspondingly, agonist-stimulated [^35^S]GTPγS binding in the PAG and spinal cord was reduced by chronic THC treatment in WT but not in β-arrestin2 KO mice, while no changes were found in the preoptic area of the hypothalamus for either genotype. These findings indicate that β-arrestin2 regulates desensitization of CB1 induced G-protein activity in PAG, spinal cord, and preoptic area of the hypothalamus, and that desensitization by β-arrestin2 is the underlying mechanism behind the development of tolerance to cannabinoid antinociception and hypothermia. Interestingly, the development of tolerance to THC catalepsy was enhanced in β-arrestin2 KO mice, and agonist-stimulated [^35^S]GTPγS binding was reduced in basal nuclei—globus pallidus and substantia nigra—after chronic THC treatment in β-arrestin2 KO but not in WT mice [103]. Since basal nuclei have been implicated in cannabinoid-induced catalepsy [113,114], these findings indicate that G-protein desensitization in CB1 receptors located in the basal nuclei confers tolerance to catalepsy, but the mechanism for tolerance development, in this case, is not due to CB1 interaction with β-arrestin2 but may instead be due to β-arrestin1.

It has been suggested that, for the CB1 receptor, β-arrestin1 and β-arrestin2 have different roles in signaling and endocytosis, with β-arrestin1 responsible for pERK1/2 signaling and β-arrestin2 responsible for receptor internalization [115]. Since β-arrestin recruitment is preceded by G-Protein-coupled receptor kinase (GRK)-mediated phosphorylation of Ser/Thr residues on the C-terminus, studies investigated the impact of mutations on the C-terminal putative GRK3 phosphorylation sites Ser426 and Ser430 on CB1 receptor desensitization, internalization, and β-arrestin-mediated signaling. The S426A/S430A CB1 receptor shows attenuated desensitization and receptor internalization [116,117]. Further, when compared to WT, S426A/S430A elicits a more prolonged pERK1/2 response, which is independent of receptor internalization but also insensitive to inhibition of Gα_i/o_ and Gα_s_ with PTx and cholera toxin, respectively [117]. Delgado-Peraza et al. [118] showed that suppressing β-arrestin1 translation eliminated 2-AG and WIN55212-2 induced pERK1/2 signaling by S426A/S430A CB1, while suppressing β-arrestin2 translation had no effect on early pERK1/2 and only partially reduced sustained pERK1/2. They also showed that at 20 min after treatment with WIN55212-2, S426A/S430A highly colocalizes with β-arrestin1, while WT CB1 does not. Further, by performing coimmunoprecipitation, they found that S426A/S430A CB1 shows greatly enhanced association with β-arrestin1 after 5 min WIN55212-2 treatment. In contrast, association with β-arrestin2 was present in WT CB1 but greatly reduced in S426A/S430A. This finding indicates that GRK3 phosphorylation at Ser426 and Ser430 (Ser425 and Ser429 in human CB1) switches the receptor’s preference from recruitment of β-arrestin1 to the recruitment of β-arrestin2. Indeed, suppressing GRK3 translation, which likely inhibits CB1 internalization, promoted sustained pERK1/2 signaling at the WT CB1 receptor [118]. The observation that S426A/S430A highly recruits β-arrestin1 instead of β-arrestin2 and shows enhanced pERK1/2 suggests that β-arrestin1 mostly mediates arrestin-dependent pERK1/2 signaling. In contrast, the finding that S426A/S430A is resistant to internalization and shows reduced β-arrestin2 recruitment indicates that β-arrestin2 mostly mediates receptor internalization. Nevertheless, the fact that CB1 downregulation, as measured by radioligand binding, still occurs in the brains of β-arrestin2 KO mice, albeit in a brain-region-specific manner [103], shows that β-arrestin1 is capable of internalization under certain conditions. Further, the fact that β-arrestin2 siRNA knockdown partially reduced pERK1/2 from S426A/S430A CB1, although only at later time points [118], shows that β-arrestin2 is capable of inducing pERK1/2 signaling to a lower extent. In conclusion, although CB1 recruits both β-arrestin1 and β-arrestin2, there are brain region- and ligand-specific differences in the roles of each of these proteins regarding CB1 internalization and signaling that may translate to different roles on cannabinoid-induced effects.

## 5. CB1-Biased Signaling

### 5.1. Orthosteric Ligands

As mentioned above, CB1 ligands show potential therapeutic effects in numerous neurological disorders. However, the development of CB1 targeted pharmacotherapeutics remains hindered by concerns about adverse effects, rapid tolerance, and abuse potential. Since CB1 can activate both heterotrimeric G-proteins and β-arrestins, novel drug discovery efforts have focused on exploring biased signaling to mitigate some of these issues while maintaining therapeutic effects, as has been reported for several other GPCR systems [119].

CB1 ligands are capable of functional selectivity, but clear biased-signaling profiles have been challenging to characterize reliably across different studies. Laprairie et al. [101] compared β-arrestin1 signaling from CB1 orthosteric agonists in a mouse striatal derived cell line (STHdh). In this study, the rank order of potency for β-arrestin1 recruitment was as follows: THC > CP55940 > WIN55212-2 » 2-AG » AEA. Efficacy for β-arrestin1 recruitment was similar among THC, CP55940, and 2-AG but lower with AEA. WIN55212-2 efficacy was lower than that of THC, CP55940, and 2-AG but did not reach statistical significance. Furthermore, pERK1/2 signaling was sensitive to PTx treatment in an early time point for AEA, 2-AG, CP55940, and WIN55212-2 but not for THC. These data indicate that THC has a more β-arrestin1 biased signaling profile than the other ligands tested, while AEA shows more sensitivity to G-protein inhibition. Since the pERK1/2 response is used to estimate G-protein signaling, affirmations on G-protein-biased signaling should be confirmed by analysis of G-protein activation, with cAMP inhibition, or [^35^S]GTPγS binding, for instance. In another study, Laprairie et al. [120] investigated CB1-biased signaling in STHdh cells expressing WT or mHTT, and calculated bias factors using the operational model [121] with WIN55212-2 as the reference ligand. When comparing the Gα_i/o_-dependent pERK1/2 response and β-arrestin1 recruitment, they found that THC and CP55940 show β-arrestin1 biased signaling, while the endocannabinoids 2-AG and AEA show Gα_i/o_ biased signaling. Since pERK was used to assess Gα_i/o_ signaling, it is necessary to exercise caution when analyzing these data, as pERK1/2 is a response that can be elicited by other G-proteins as well as by β-arrestins, which may be a confounding factor. In addition, β-arrestin2 recruitment was not investigated since STHdh cells do not express β-arrestin2. Nonetheless, CP55940 and THC were detrimental to cell viability, while 2-AG, AEA, and WIN55212-2 improved viability in cells expressing mHTT. This finding suggests that CB1 G-protein signaling is neuroprotective in HD.

In a different study, Khajehali et al. [122] investigated cAMP inhibition and pERK1/2 in Chinese hamster ovary (CHO) cells stably expressing human CB1 receptor and calculated the bias factor using the operational model with 2-AG as the reference ligand. In this case, WIN55212-2 showed a similar signaling profile to 2-AG, while CP55940, THC, and AEA showed a tendency toward cAMP inhibition bias, although that difference was not statistically significant. HU-210 and methanandamide, on the other hand, showed a significant bias toward cAMP inhibition. Since this study did not assess PTx sensitivity or β-arrestin recruitment, it is difficult to ascertain the origin of the pERK1/2 response and whether it could be used to estimate relative levels of β-arrestin bias.

More recently, Zhu et al. [123] evaluated cAMP inhibition, pERK1/2 response and receptor internalization in HEK293 cells stably expressing human CB1 receptor and calculated ligand bias factors using a kinetic model with 2-AG as the reference ligand. In this study, WIN55212-2 also showed a similar signaling profile to 2-AG. On the other hand, THC showed a strong bias toward pERK1/2 and receptor internalization over cAMP inhibition, while CP55940 and AEA showed bias toward receptor internalization but only moderate bias toward pERK1/2. These findings suggest that CP55940, AEA, and THC show a β-arrestin-biased signaling profile; however, this should be confirmed by β-arrestin recruitment assays. As previously mentioned, pERK1/2 can be stimulated by multiple transducers, and although receptor internalization is generally a good proxy for β-arrestin recruitment, it is possible that β-arrestin1 and β-arrestin2 exert different functions, in which case, ligand-specific preference for either β-arrestin could be a confounding factor.

In contrast, Ibsen et al. [100] investigated CB1 mediated β-arrestin1 and β-arrestin2 translocation to the plasma membrane in HEK293 cells with different results. In this study, only 2-AG and WIN produced an amount of β-arrestin1 translocation that was different from control. In β-arrestin2 translocation; however, the rank order of potency was CP55940 > WIN55212-2 > AEA > 2-AG, while the rank order of efficacy was 2-AG > WIN55212-2 > CP55940 > AEA. In this case, THC did not significantly stimulate β-arrestin2 translocation. All in all, the studies that have sought to compare ligand bias among orthosteric CB1 agonists have failed to reliably identify biased ligands, with conflicting results under different experimental conditions, even in the same cellular background. This could indicate that all of these ligands are relatively balanced when it comes to shifting the conformational dynamics to a state that favors G-protein coupling or a state that favors β-arrestin recruitment, and that strongly biased CB1 orthosteric ligands have not yet been described. Understanding the molecular mechanism behind biased signaling will be of paramount importance for the design of novel CB1 ligands with a better biased-signaling profile.

### 5.2. Allosteric Ligands

**Figure 2:**
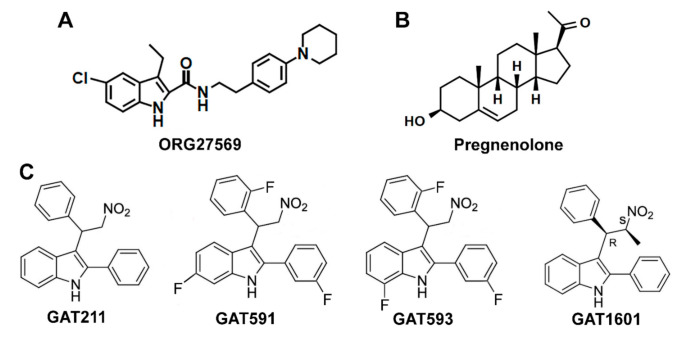
Biased allosteric ligands of CB1: (**A**) molecular structure of ORG27569; (**B**) molecular structure of pregnenolone; (**C**) molecular structures of GAT211 and its analogs, two fluorinated analogs—GAT591 and GAT593—and one methylated analog—GAT1601.

**Figure 3:**
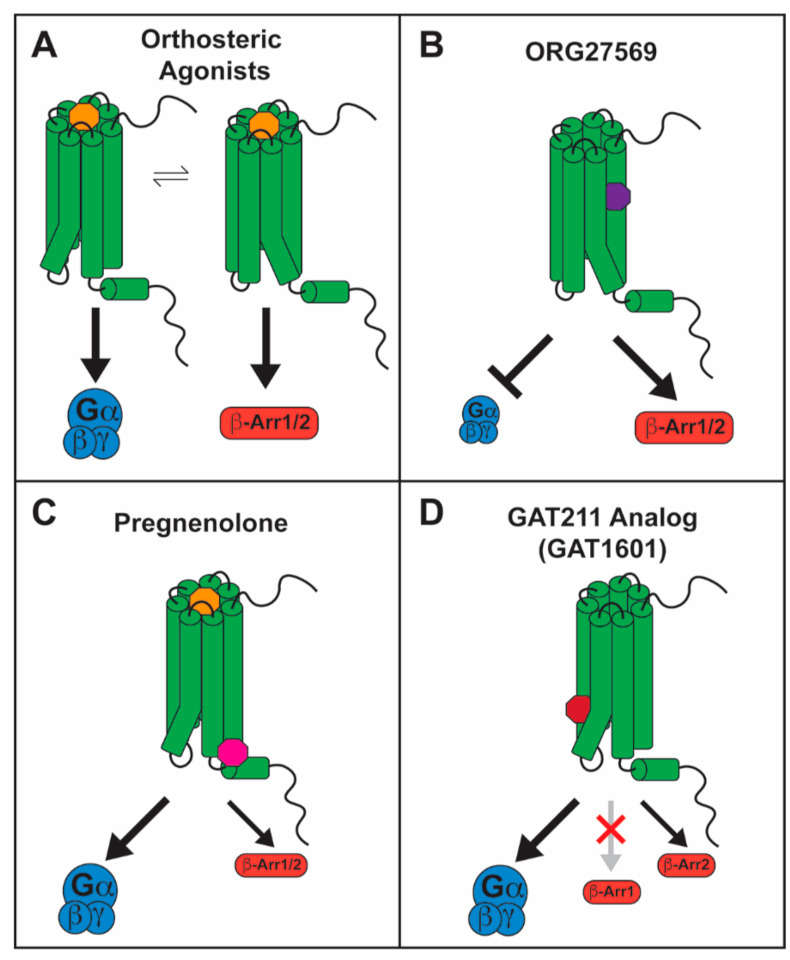
Biased signaling by CB1 allosteric ligands: (**A**) orthosteric ligands, represented in yellow, promote G-protein signaling and β-arrestin recruitment in a balanced manner, stimulating conformational changes important for both; (**B**) ORG27569, in purple, inhibits G-protein signaling and stimulates β-arrestin signaling, whether alone or in the presence of an orthosteric ligand, generating β-arrestin-biased signaling. This occurs due to inhibition of TMH6 movement and stimulation of TMH7/Hx8 movement, respectively; (**C**) pregnenolone, in magenta, inhibits β-arrestin signaling in the presence of an orthosteric ligand, generating G-protein-biased signaling. This occurs due to inhibition of TMH7/Hx8 movement while allowing TMH6 movement; (**D**) GAT1601, in dark red, stimulates G-protein signaling and β-arrestin2 recruitment to a smaller extent but not β-arrestin1 recruitment, generating G-protein-biased signaling. This occurs due to the facilitation of TMH6 movement.

Orthosteric agonists, antagonists, and inverse agonists bind to the primary, orthosteric, binding pocket and compete for binding with endogenous ligands. On the other hand, allosteric ligands bind to an allosteric site, which is topologically distinct from the orthosteric binding pocket and do not compete for binding with orthosteric/endogenous ligands [124]. A ligand can be a negative allosteric modulator (NAM), inhibiting signaling from an orthosteric agonist, or a positive allosteric modulator (PAM), enhancing signaling from an orthosteric agonist. Neither NAMs nor PAMs produce signaling in the absence of an orthosteric agonist. However, some ligands are allosteric agonists, promoting signaling in the absence of an orthosteric ligand, and some compounds can have both PAM and allosteric agonist effects (ago-PAM), inducing signaling when administered alone, as well as potentiating signaling from an orthosteric agonist. Pharmacologists are increasingly seeking allosteric ligands as a strategy to develop improved small molecule therapeutics to target GPCRs. These drugs may produce fewer side effects, given that NAMs and PAMs can alter signaling from endogenous agonists in a time-specific and site-specific manner. Another advantage is that amino acid residues in allosteric sites are less conserved across different GPCRs, which would contribute to target specificity. Further, some allosteric modulators can confer biased signaling properties to otherwise balanced agonists [124]. Endogenous and exogenous allosteric modulators have been described for the CB1 receptor (Figure 2). Some of these allosteric ligands display a biased signaling profile. Since orthosteric agonists promote balanced levels of G-protein signaling and β-arrestin recruitment (Figure 3A), the mechanism of action of biased allosteric ligands may shed light on the conformational changes that are required for CB1 mediated β-arrestin-biased signaling and make allosteric binding pockets better candidates for the development of novel CB1 biased ligands.

#### 5.2.1. ORG27569 as a Biased Allosteric Modulator of CB1

The first allosteric modulator described for the CB1 receptor was ORG27569 (ORG), a 1*H*-indole-2-carboxamide analog (Figure 2A) that was first described as a NAM for CB1. ORG was found to enhance binding and slow the dissociation rate of CP55940 but inhibit G-protein activation [125,126,127]. ORG also antagonized inhibition of cAMP by CP55940, WIN55212-2 and AEA [126,128]. In the absence of an orthosteric agonist, ORG inhibited CB1 constitutive activity [126,127]. These findings indicate that ORG promotes desensitization of G-protein signaling at the CB1 receptor. In accordance, ORG inhibited DSE in primary hippocampal neurons [129], indicating that it can also negatively regulate 2-AG mediated G-protein signaling.

Remarkably, ORG enhanced CP55940-mediated pERK1/2 signaling, and also stimulated this signaling pathway when administered alone [115,126,127]. This effect was abolished by suppressing β-arrestin1 translation but not by suppression of β-arrestin2 [115]. Further, β-arrestin1 colocalized with CB1 receptor under fluorescence microscopy after treatment with ORG [115]. Agonist-induced receptor internalization and β-arrestin2 recruitment, however, were inhibited by ORG [126,128,130]. In contrast, Ahn et al. [115,127] reported increased receptor internalization via a β-arrestin2-dependent mechanism when ORG was administered alone. This disparity could be attributed to the fact that Ahn et al. used a mutant CB1 receptor (T210A) for their internalization assays to enhance the presence of CB1 on the plasma membrane at baseline. The enhanced presence of CB1 on the membrane or differences in the receptor structure caused by the mutation could impact the way ORG influences CB1 signaling, switching from β-arrestin1 to β-arrestin2 recruitment. Likewise, differences in GRK expression levels among different in vitro experimental systems could affect ORG-induced β-arrestin recruitment, where preference for β-arrestin1 results in ORG-stimulated pERK1/2 signaling and reduced internalization, but no discrimination between β-arrestin isoforms leads to stimulation of both pERK1/2 and internalization. A similar mechanism could also explain divergent results from different labs, where ORG enhanced [115,126,127] or inhibited [122,130] agonist-stimulated pERK1/2. If ORG preferentially recruits β-arrestin1, differences in GRK isoform expression or β-arrestin1 expression levels may occlude β-arrestin1 pERK1/2 signaling and produce an antagonistic effect on G-protein mediated pERK1/2 signaling. All in all, the body of evidence suggests that ORG is a β-arrestin biased ligand at the CB1 receptor, functioning as an allosteric inverse agonist and NAM for G-protein signaling and an allosteric agonist and PAM for β-arrestin1-mediated pERK1/2 signaling (Figure 3B).

Studies on the impact of ORG on receptor conformation have begun to shed light on the molecular mechanism for β-arrestin recruitment and signaling at the CB1 receptor. Using site-directed fluorescence labeling, Fay and Farrens [86] showed that ORG enhances conformational changes at TMH7/Hx8, in the absence of TMH6 movement. Although this is an important finding, biophysical methods such as these are unable to define conformational changes in such a way as to determine a molecular mechanism. Recently, a crystal structure of CB1 bound to ORG and CP55940 was reported [131]. In this structure, the outward movement of TMH6 that would normally be induced by an orthosteric agonist such as CP55,940, was greatly inhibited [131], which explains the NAM effect of ORG on CP55,940 mediated G-protein signaling [125]. Further, a slight outward movement of the intracellular domain of TMH7 is seen in ORG- and CP55940-bound CB1, when compared to MDMB-FUBINACA-bound CB1 [76,131]. However, the presence of five thermostabilizing mutations (T210A, E273K, T283V, R340E, and S203K) within the transmembrane helices and a truncated C-terminus that were applied to aid in crystallization [131] may affect overall receptor structure and preclude observation of further conformational changes and intramolecular interactions that may be induced by ORG on the TMH7/Hx8 elbow. Further, ORG was shown to also induce β-arrestin signaling in the absence of CP55940 [115,127]. Since the CB1 crystal structure was obtained with both ORG and CP55940 [131], how ORG may affect CB1 conformational dynamics in the absence of an orthosteric agonist is still poorly understood. Using MD simulations, Lynch et al. [132] indicated that after ORG binds to CB1, it promotes an outward movement of the IC domain of TMH7. This does not open the site for interaction with the G-protein at the TMH3/5/6 region but opens a site for interaction with β-arrestin at the TMH7/1/2 region. However, the findings have yet to be confirmed experimentally. These studies attribute to the TMH7/Hx8 region the role of promoting the alternative active state of CB1 that promotes β-arrestin-biased signaling. 

When administered in vivo, ORG had no effect on CP55940-mediated antinociception or catalepsy [133,134]. Interestingly, ORG had no effect on CP55940-mediated hypothermia in C57BL/6J mice but attenuated this response in Sprague Dawley rats [133,134]. Further, ORG reduced AEA-induced hypothermia [134], an effect that is not blocked by the selective CB1 inverse agonist SR141716A [135,136], indicating that ORG antagonizes AEA hypothermia via a non-CB1 mechanism. Interestingly, ORG administered alone decreases body weight and food intake in mice [133] and rats [134]. However, this effect was also observed in mice with genetic deletion of CB1, indicating that the effect is not mediated by CB1. These findings suggest that ORG has at least one non-CB1 target in vivo, and this could be an additional possible explanation for diverging results with ORG in different studies.

#### 5.2.2. Pregnenolone as a Biased Allosteric Modulator of CB1

An endogenous NAM for CB1 has also been described. Pregnenolone, 3α-hydroxy-5β-pregnan-20-one (Figure 2B), is a steroid hormone that was found to be a signaling specific NAM for CB1 [137]. Exposure to THC upregulates pregnenolone synthesis via a pERK1/2-induced increase in the levels of cytochrome P450scc [137]. Pregnenolone then antagonizes the effects of THC on synaptic transmission and on the cannabinoid tetrad, forming a negative feedback loop. The CB1 signaling profile of THC in the presence of pregnenolone was evaluated in vitro, showing that pregnenolone effectively antagonizes THC-mediated pERK1/2 signaling and suppression of cellular and mitochondrial respiration, without influencing cAMP inhibition. Using the Force-Biased Metropolis Monte Carlo (MMC) simulated annealing program, Vallée et al. [137] showed that the pregnenolone binding site on CB1 lies on the cytoplasmic end, where pregnenolone forms hydrogen bonds with Glu133^1.49^ and Arg409^7.65^. This was confirmed by mutational analysis, as in an E133G CB1 mutant, pregnenolone has no effect on THC-mediated suppression of cellular respiration [137]. Binding at this site would tether TMH7 near TMH1, restricting conformational changes on TMH7 that are believed to be important for the β-arrestin-biased signaling state [86,132]. These findings are consistent with a role for pregnenolone as a biased NAM for CB1 β-arrestin signaling (Figure 3C). However, changes to β-arrestin recruitment in the presence of pregnenolone should be directly measured to confirm this effect.

In rodents, pregnenolone prevented THC-induced increases in food intake and memory impairment. Further, neuronal firing in the ventral tegmental area and dopamine release in the nucleus accumbens induced by THC were reduced by pregnenolone. Accordingly, pregnenolone also reduced WIN55212-2 self-administration [137]. These findings indicate that CB1-biased signaling mediated by pregnenolone may be useful for the treatment of *Cannabis* intoxication and to reduce *Cannabis* abuse potential. Importantly, pregnenolone was also shown to block a cannabinoid-induced psychotic-like state in mice [138]. As medicinal and recreational *Cannabis* become more popular, pregnenolone or potential novel analogs could become an important tool in the clinic. However, its efficacy for inhibiting signaling from endogenous cannabinoids has not yet been investigated and could be a source of adverse effects.

#### 5.2.3. GAT211 as a Positive Allosteric Modulator of CB1

GAT211 is a compound derived from 2-phenylindole (Figure 2C) that has been described as an allosteric ligand for the CB1 receptor [139]. GAT211 increased binding and slowed the dissociation rate of CP55940 from CB1 and reduced the binding of SR141716A. In functional assays, GAT211 enhanced the effect of CP55940, 2-AG, and AEA on both G-protein signaling and β-arrestin1 recruitment to similar degrees. When compared to β-arrestin2 recruitment, on the other hand, GAT211 significantly favored cAMP inhibition in CHO cells [140], suggesting a G-protein-biased signaling profile when using CP55940 as a reference ligand. In the absence of an orthosteric agonist, GAT211 is also capable of eliciting G-protein signaling and β-arrestin1 recruitment, demonstrating an ago-PAM effect at CB1. GAT211 is a racemic mixture of GAT228 (R-(+) enantiomer) and GAT229 (S-(-) enantiomer). Interestingly, GAT229 is responsible for the PAM effect on agonist-mediated signaling and shows no effect when administered alone, while GAT228 stimulated signal transduction on its own, showing an allosteric agonist profile [139]. In hippocampal neurons in vitro, GAT228 inhibited excitatory postsynaptic currents (EPSCs) on its own, further demonstrating its allosteric agonist effect [141]. While GAT229 had no effect on EPSCs alone, it enhanced DSE, supporting its role as a PAM for endocannabinoid signaling [141]. This enantiospecific effect is possible because GAT228 and GAT229 likely bind to two different allosteric sites on the CB1 receptor. Using Force-Biased MMC Simulated Annealing, Hurst et al. [142] found that GAT228 binds at an IC exosite, forming interactions with residues on TMH1, TMH2, TMH4, and ICL1, while GAT229 binds at an EC site, forming interactions with residues on TMH2, TMH3, and ECL1. These findings support the existence of separate allosteric agonist and PAM binding sites for GAT211 enantiomers.

The therapeutic potential of GAT211 and its enantiomers has been shown in several preclinical models. GAT211 and enantiomers enhanced cell viability in a striatal cell line expressing mHTT, and improved motor coordination and prevented motor impairment in the R6/2 mouse model of HD [143]. In a preclinical model of glaucoma, GAT229 reduced intraocular pressure [144]. GAT211 and enantiomers also reduced seizures in a preclinical model of childhood epilepsy [145]. Further, GAT211 induced antinociception in preclinical models of inflammatory and neuropathic pain without affecting motor coordination or body temperature, and without inducing tolerance, conditioned place preference, or antagonist precipitated withdrawal symptoms [146]. Additionally, in a preclinical model of neuropathic pain, GAT211 enhanced morphine analgesia and prevented opioid tolerance development [147]. These studies suggest that a CB1 ago-PAM, such as GAT211, could have therapeutic effects in the contexts of HD, epilepsy, and pathological pain. Importantly, GAT211 could be useful as an opioid-sparing treatment, which is especially relevant in the face of the current opioid epidemic. GAT211, as a CB1 ago-PAM, may also have therapeutic potential in disorders associated with an impaired endocannabinoid system, such as during aging and neurodegeneration, as it would be able to counteract reduced CB1 expression and boost endogenous cannabinoid signaling [24,25,26,148].

However, GAT211 is a probe compound not intended to be developed for the clinic, due to its low affinity for CB1 and rapid metabolic clearance. To address these issues, fluorinated analogs of GAT211 were developed—GAT591 and GAT593 (Figure 2C). These showed significantly enhanced potency and greater metabolic stability as measured by a microsomal stability assay [140]. The analogs did not improve upon the moderate G-protein-biased signaling profile of GAT211 but maintained a similar slight preference for cAMP inhibition over β-arrestin2 recruitment, compared to CP55940 [140]. When administered in vivo, the fluorinated analogs suppressed mechanical allodynia in a preclinical model of inflammatory pain with a much longer duration of action than previously reported for GAT211 [140], likely due to enhanced metabolic stability. Remarkably, the fluorinated analogs also produced antinociception in naïve mice, without affecting catalepsy or hypothermia [140], which are frequently observed with orthosteric agonists. This may be attributed to the preference for G-protein signaling over β-arrestin2 recruitment since β-arrestin2 KO studies suggest that cannabinoid antinociception is hindered, while catalepsy is enhanced by the presence of β-arrestin2 [103], as previously discussed. Interestingly, methylated GAT211 analogs modified biased signaling in a diastereomer-specific manner. One diastereomer, GAT1601 (Figure 2C), was an effective ago-PAM for cAMP inhibition and β-arrestin2 recruitment, but it did not enhance β-arrestin1 recruitment [149]. In a preclinical model of glaucoma, this compound was more effective and had a longer-lasting effect than the more balanced diastereomer or the parent compound GAT211 [149]. These results suggest that this “anti-β-arrestin1” signaling bias (Figure 3D) may also present a therapeutic advantage in some pathological conditions.

## 6. Conclusions

CB1 is a GPCR that signals primarily via G_i/o_ proteins. However, signaling promiscuity is reported throughout the literature. In addition to G_i/o_, CB1 has been shown to couple to G_s_ [92], G_q/11_ [96], and G_12/13_ [98]. Although the fraction of non-G_i/o_ protein activation is reportedly small, it is possible that activation of different G-protein subtypes is associated with different CB1 functions. For instance, while G_i/o_ is responsible for the suppression of synaptic neurotransmitter release [44], G_12/13_ activation may be responsible for CB1 mediated regulation of neurite growth [99]. Consequently, this poorly studied aspect of CB1 signaling could have a fundamental impact on the role of CB1 during brain development. How non-G_i/o_ signaling affects cannabinoid-induced physiological effects must be studied further to ascertain whether shifting G-protein subtype preference could pose a therapeutic advantage when targeting CB1. Regardless of which G-protein is coupled by CB1, the mechanism of activation culminates in an outward movement of the intracellular domain of TMH6, while the G-protein subtype flexibility is likely due to weak interactions of the receptor ICL2 with the G_α_ on the intracellular surface [76]. Mutations on the ICL2, therefore, may increase or decrease CB1 mediated signaling via non-G_i/o_ proteins, which could elucidate the role of these signaling pathways on cannabinoid function. This activation mechanism is now well understood for CB1 orthosteric agonists and it shares similarities with other class A GPCRs [150,151,152].

On the other hand, the mechanism and function of β-arrestin recruitment by CB1 is less well understood. At the functional level, β-arrestins regulate CB1 desensitization and downregulation in a brain-region-specific manner, which can result in differential tolerance development to cannabinoid effects [103]. An important distinction seems to exist between the functions of β-arrestin1 and β-arrestin2, where cannabinoid antinociception may be enhanced by β-arrestin1 [105] but hindered by β-arrestin2 [103,112]. Further, switching between β-arrestin1 and β-arrestin2 via the absence or presence of phosphorylation by GRK3 affects early and sustained pERK1/2 responses [118], which could have different roles on the downstream effects of CB1 activity. However, little is known about the consequences of favoring CB1 recruitment of β-arrestin1 or β-arrestin2. Most known agonists stimulate both β-arrestins and specificity may stem from a cell-specific context. One recently discovered exception is GAT1601, which enhances β-arrestin2 recruitment but not β-arrestin1 [149]. Interestingly, this compound showed stronger therapeutic potential than more balanced compounds in a preclinical model of glaucoma, suggesting that dissociating β-arrestin1 from β-arrestin2 recruitment could be beneficial when targeting CB1 in this context. More studies are required to better understand the differences between the functions of both β-arrestins downstream of CB1 activation under different cellular backgrounds so this potential can be exploited in CB1 targeted therapeutics.

The biased-signaling properties of CB1 orthosteric agonists have been investigated in different in vitro systems. However, common CB1 ligands show little preference for either G-protein or β-arrestin signaling, with conflicting results across different studies [100,101,120,122,123]. This suggests that classical cannabinoids are fairly unbiased and that differential signaling depends largely on the cellular background. Allosteric CB1 ligands, on the other hand, have been successfully used to selectively trigger or inhibit specific signal transducers [115,126,127,137,140,149]. This suggests that allosteric-binding sites hold better promise for the development of strongly biased CB1 ligands. In fact, their proposed mechanism of action is to stimulate or inhibit conformational changes on the TMH7/Hx8 elbow of the receptor to stimulate or inhibit β-arrestin signaling [86,132,137], which is consistent with the putative molecular mechanism of β-arrestin-biased signaling [84,86,87,88,153]. As an exception, GAT211 and analogs do not affect the conformation of TMH7 but instead facilitate the movement of TMH6 [142], leading to G-protein-biased signaling. In fact, when administered alone, in agonist mode, GAT211 analogs showed little to no stimulation of β-arrestin recruitment [149]. As allosteric agonists, these compounds bind to a TMH1-2-4 exosite, stimulating the movement of TMH3 toward TMH4 and stretching the ionic lock until it is broken, which facilitates the outward movement of TMH6 [142]. Therefore, exploration of this allosteric agonist site can potentially produce novel strongly G-protein-biased CB1 agonists.

In conclusion, CB1 is highly expressed throughout the CNS in excitatory and inhibitory neurons as well as astrocytes, giving it the potential to impact a myriad of CNS physiological functions and disease states. However, this broad expression also limits its utility due to adverse effects and abuse potential. Biased signaling has been suggested as a strategy to dissociate therapeutic effects from the undesired effects of CB1 activity. However, the functional consequences of CB1-biased signaling are still poorly understood due to the lack of signaling specificity of know orthosteric agonists. The development of biased allosteric ligands may be a viable strategy to dissociate the activation of G-proteins, β-arrestin1, or β-arrestin2 and refine CB1 targeted therapeutics.

## Data Availability

Not applicable.
